# Improved Immune Moth–Flame Algorithm for Intelligent Vehicle Parking Path Optimization

**DOI:** 10.3390/biomimetics11040245

**Published:** 2026-04-03

**Authors:** Yan Chen, Longda Wang, Xiujiang Zhu, Gang Liu

**Affiliations:** 1 School of Mechanical and Electrical Engineering, Chizhou University, Chizhou 247000, China; 2 School of Electrical Engineering, Dalian Jiaotong University, Dalian 116026, China; 3 College of Engineering, Inner Mongolia Minzu University, Tongliao 028000, China

**Keywords:** intelligent trajectory optimization, cubic spline interpolation, parking trajectory optimization, moth–flame algorithm, immune mechanism

## Abstract

Intelligent parking systems have been recognized as a core technological intervention for resolving parking garage shortages and advancing traffic safety. Nevertheless, it remains challenging to generate a smooth, accurate, and optimal parking trajectory when employing conventional intelligent path optimization algorithms. Hence, building upon a newly designed optimization model for intelligent vehicle parking path planning, this study develops an improved immune moth–flame optimization algorithm (IIMFO). Specifically, aiming at the shortest path length and smooth enough trajectory, we leverage a cubic spline interpolation-driven path planning model to resolve the complex automatic parking trajectory optimization problem. To significantly strengthen the optimization effect, we introduce immune concentration selection, nonlinear decaying adaptive inertia weight adjustments, and elite opposition-based learning mechanisms to improve the immune moth–flame algorithm. Based on the evaluation results of the test functions, as well as the simulation and semi-automatic experiments of the real-world scenario of intelligent vehicle parking path optimization, the results indicate that the improved strategy can achieve better parking trajectories.

## 1. Introduction

With the surge in urban vehicle count and the steady decline in available parking garages, intelligent parking systems have been recognized as a core technological intervention for resolving parking garage shortages and advancing traffic safety. Path planning serves as a decision core module in intelligent parking systems, tasked with dynamically generating admissible trajectories that meet the vehicle’s motion limitations, guarantee collision-free navigation, and maintain curvature continuity. Recently, there has been growing academic interest in developing path planning methods that simultaneously achieve high computational efficiency and smooth trajectories, especially under limited parking areas and crowded scenes [1].

In contrast to traditional geometric curve approaches, search-based methods have gained broader adoption for addressing intricate planning tasks. Notably, Zhang et al. developed a trajectory planning approach that synergistically incorporates Hybrid A* with nonlinear model predictive control (NMPC) [2]. And Vlasak et al. lowered the computational expense by improving the expansion strategy of RRT [3]. Nevertheless, paths initially produced by these search-based methods often exhibit discontinuity, thereby calling for dedicated smoothing procedures to yield kinematically viable trajectories.

To further enhance the smoothness and ride comfort of the path trajectories, spline-based interpolation techniques have become the mainstream approach. Sim et al. proposed a mesh path planning algorithm based on kinematic constraints, utilizing cubic spline interpolation to generate high-quality trajectories with continuous curvature [4]. Similarly, Lian et al. developed a path planning strategy for mobile robots utilizing cubic spline interpolation and demonstrated its stable performance under dynamic environmental conditions [5]. In addition, Ye et al. introduced a trajectory generation approach for intelligent parking applications, leveraging B-spline curves to ensure smoothness in both longitudinal motion and steering actuation [6]. The above indicates that applying spline theory to parking trajectory modeling can effectively meet the continuity requirements of vehicle steering.

Metaheuristic approaches are extensively utilized in path parameter optimization, primarily because of their exceptional ability to explore the global search effectively. Conventional optimization techniques, including Particle Swarm Optimization (PSO) [7], Grey Wolf Optimizer (GWO) [8], and Whale Optimization Algorithm (WOA) [9], are commonly employed across various domains. Zhu et al. also verified the advantages of algorithms based on concentration selection strategies in dynamic obstacle avoidance [10]. However, according to the “No Free Lunch” theorem [11], their tendency to converge prematurely to local optimal solutions remains a significant limitation. To this end, Mirjalili’s Moth–Flame Optimization (MFO) algorithm has proven highly effective for tackling complex engineering optimization tasks, largely owing to its characteristic logarithmic spiral-based search strategy for solution refinement [12,13]. In recent years, extensive scholarly attention has been directed toward refining the MFO algorithm. Building on these advancements, Dai et al. integrated an enhanced MFO into mobile robot navigation systems and validated its effectiveness in generating high-quality paths amid densely distributed obstacles [14]. Chen et al. introduced a targeted mutation strategy to enhance the population diversity of MFO, thereby solving the premature convergence problem in the reversing parking task [15].

Although the remarkable achievements have been brought about through the aforementioned improvements, in the high-dimensional and complex intelligent parking scenarios, the MFO algorithm still faces challenges such as slow convergence speed and a high tendency to get trapped in local optima. To address these limitations, incorporating principles derived from the biological immune system has emerged as an effective enhancement strategy. Liu et al. developed a novel hybrid optimization approach that combines the immune system with the moth–flame algorithm, incorporating gene correction operators to refine solution quality, thereby boosting the precision of intelligent parking path generation [16]. Furthermore, Zhao et al. proposed a multi-objective optimization strategy based on r-domination, offering a novel approach for achieving an effective trade-off between the algorithm’s exploratory and exploitative capabilities [17]. Existing studies also indicate that introducing an adaptive inertia weight mechanism and the clonal selection mechanism from artificial immune systems are effective means to maintain population diversity and suppress premature convergence [18,19].

Motivated by the aforementioned challenges in conventional intelligent trajectory control systems, this study develops a novel path planning framework. Specifically, an intelligent vehicle parking path optimization model utilizing cubic spline interpolation is formulated. Concurrently, an improved immune moth–flame optimization (IIMFO) algorithm is proposed to fundamentally resolve the dual challenges of excessively long parking paths and discontinuous tracking trajectories, while successfully overcoming the conventional MFO algorithm’s inherent susceptibility to premature convergence.

Relative to previous work on optimizing trajectories for intelligent parking, this study presents the following novel contributions:

(I) Key advancement in parking path optimization: To resolve the dual challenges of unnecessarily long parking paths and discontinuous tracking trajectories inherent in traditional optimization-based approaches, this study develops a new path generation strategy grounded in cubic spline interpolation. This strategy effectively converts the high-dimensional, nonlinear trajectory optimization task into a computationally efficient and smooth feasible solution.
(II) Key advancement in MFO: To address the susceptibility of conventional intelligent optimization algorithms to premature convergence, this paper presents an improved immune moth–flame optimization (IIMFO) algorithm that explicitly incorporates escape mechanisms to overcome MFO’s inherent limitation in escaping local optima. Three significant modifications form the core of this improved framework, elaborated as follows: I integrate an immunity-based operator into the iterative optimization cycle to enrich solution diversity and extend the exploratory range; II implement weight coefficients that decrease adaptively over iterations, thereby enhancing the adaptability of the algorithm in different search stages; III deploy opposition-based learning (OBL) on the elite population to elevate solution precision and strengthen global optimization efficacy. The numerical results of the test functions and the simulation and semi-automatic experiment results of intelligent vehicle parking path optimization operational examples indicate the improved strategies can significantly enhance optimization speed and accuracy.

This paper is structured as follows. Section 2 depicts the path optimization method using cubic spline interpolation. Section 3 describes the foundational MFO and comprehensively presents the proposed IIMFO algorithm. Section 4 details the research methodology and experimental setup. Section 5 evaluates the IIMFO through benchmark test functions, simulations, and real-world semi-automatic experiments, alongside an extended discussion. Section 6 concludes this study.

## 2. Path Planning Approach for Intelligent Parking Systems Leveraging Cubic Spline Interpolation

### 2.1. Cubic Spline Interpolation

The studies about cubic spline interpolation began in 1960s. The setting spline workers fixe the elastic slender wooden strip on several similar points using pressure iron and referring the fixed curve function table, while allowing them to freely bend at other positions. The curve formed by them is called spline curve [20].

The specific function about cubic spline interpolation is defined as follows: It is assumed that the closed interval $\Delta =\left[a,b\right]$ contains *n* + 1 distinct interpolation nodes $\left(x_{0},x_{1},\cdots \cdots ,x_{n}\right)$. The function *s*(*x*) is defined as the cubic spline interpolation function associated with the interval Δ if it satisfies the following two conditions.

**Condition** **1.** *For all subintervals $\left[x_{i},x_{i+1}\right]$, where i∈[0,1,2,…,n], s(x) can be expressed as a cubic polynomial. The specific cubic polynomial is as described in Equation (1):*

(1) $$s\left(x\right)=\left\{\begin{array}{c} s_{1}\left(x\right)=a_{1}x^{3}+b_{1}x^{2}+c_{1}x+d_{1}x\in \left[x_{0},x_{1}\right]\\ s_{2}\left(x\right)=a_{2}x^{3}+b_{2}x^{2}+c_{2}x+d_{2}x\in \left[x_{1},x_{2}\right]\\ \ldots .\\ s_{n}\left(x\right)=a_{n}x^{3}+b_{n}x^{2}+c_{n}x+d_{n}x\in \left[x_{n-1},x_{n}\right] \end{array}\right.$$

*
where $\left(x_{0},x_{1},\cdots \cdots ,x_{n}\right)$ is interpolation points; $\left(a_{0},a_{1},\cdots \cdots ,a_{n}\right)$, $\left(b_{0},b_{1},\cdots \cdots ,b_{n}\right)$, $\left(c_{0},c_{1},\cdots \cdots ,c_{n}\right)$ and $\left(d_{0},d_{1},\cdots \cdots ,d_{n}\right)$-correspond to the coefficients defining the piecewise function of cubic spline interpolation.*

**Condition** **2.** *In the closed interval $\Delta =\left[a,b\right]$, both the spline function s(x) and its first derivative $s^{\prime }\left(x\right)$ are continuous.*

### 2.2. Intelligent Vehicle Parking Path Optimization Model

**Figure 1:**
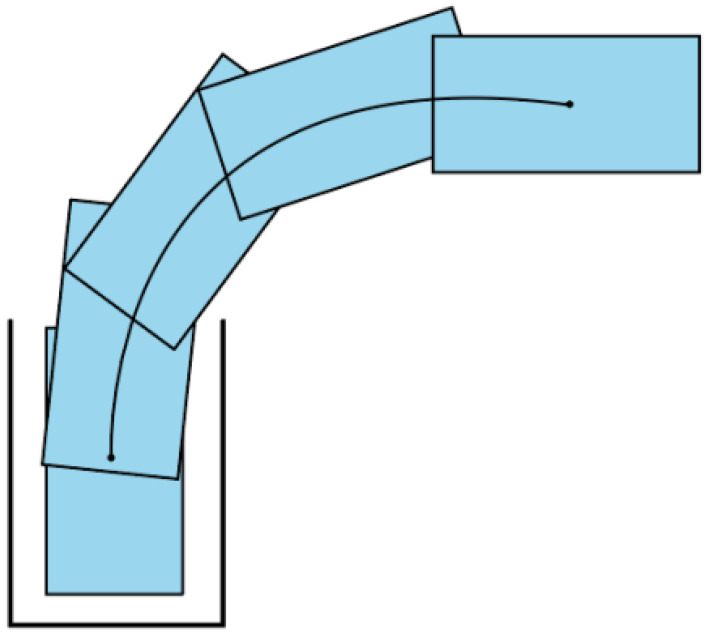
Schematic diagram of intelligent vehicle parking path optimization.

There are many forms for intelligent trajectory optimization, among them, reverse trajectory optimization is a usual vehicle parking scene. Figure 1 presents a detailed schematic illustrating the optimization process for parking trajectories in intelligent vehicles.

As depicted in Figure 1, the garage’s boundary line and the corner are distinctly visible and readily recognizable. The vehicle begins the parking process from a fixed initial position and must complete parking at the designated target location. Concurrently, the intelligent trajectory optimization and control module acquires environment and real-time relative positioning data and verifies compliance with the established parking requirements. Provided that all parking requirements are satisfied, the intelligent trajectory parking system leverages acquired environmental and vehicle state data to plan an optimal parking trajectory and execute high-precision path following, enabling accurate parking at the designated parking zone.

The proposed optimization model for intelligent vehicle parking focuses on reducing the total path length required to reach a designated parking zone; hence, it prioritizes path length minimization as its core objective, where the solution is determined by several parking location reference points. Meanwhile, it is governed by four essential conditions: an optimal parking trajectory is generated; the parking reference point is selected from a pre-established candidate set, which is derived from sensor-collected environmental data; crucially, the trajectory must satisfy collision-avoidance constraints with respect to the garage boundary lines. The overall intelligent vehicle parking path optimization problem is formally formulated as follows:

(2) $$\begin{array}{c} \min L=\int_{0}^{T_{\max}}v(t)dt\approx \sum_{it=1}^{n_{t}}\Delta L_{it}\\ S.T.\left\{\begin{array}{c} LT_{X,Y}=s\left(p_{1},p_{2},\ldots ,p_{is},\ldots ,p_{n_{s}}\right)\\ \Delta L_{it}\leq \Delta L_{\max}\&\Delta {∠}_{it}\leq \Delta {∠}_{\max}\\ \forall p_{is}\in P_{C}\\ \forall P_{g}\left(i\right)\notin \Omega_{c,it}\\ \forall P_{g}\left(i\right)\in \Omega_{g} \end{array}\right. \end{array}$$

where *P_g_* refers to the set of position for collision detection points around the garage, the number of the set *P_g_* is *n_g_*, $P_{g}\left(i\right)$ is the position of *i*-th garage collision detection point, $i\in \left\{1,2,\ldots ,n_{g}\right\}$; $\Delta L_{it}$ and $\Omega_{c,it}$ are the *i*-th time period parking interval distance and vehicle coverage area, the number of distinct time period is *n_t_*, $it\in \left\{1,2,\ldots ,n_{t}\right\}$, thus, parking termination time $T_{\max}=n_{s}\Delta t$, due to vehicles must collision avoidance garage boundary line, $\forall P_{g}\left(i\right)\notin \Omega_{c,it}$; *L* is length of parking path trajectory, it is the definite integral parking velocity v(t) about *t* from parking begin 0 to parking termination time $T_{\max}$, approximately equal to the sum of the parking interval distances for all time periods; $\Delta L_{\max}$ and $\Delta {∠}_{\max}$ are the maximum value of parking interval distance and parking attitude angle for any allowed time period; $\Delta {∠}_{it}$ are the *i*-th time period parking attitude angle, $\Delta L_{it}\leq \Delta L_{\max}\&\Delta {∠}_{it}\leq \Delta {∠}_{\max}$; $LT_{X,Y}$ is the parking trajectory in the plane coordinate system composed of *X* axis and *Y* axis; $\left\{p_{1},p_{2},\ldots ,p_{is},\ldots ,p_{n_{s}}\right\}$ is the reference point set for parking location identification, $is\in \left\{1,2,\ldots ,n_{s}\right\}$, the number of set $\left\{p_{1},p_{2},\ldots ,p_{is},\ldots ,p_{n_{s}}\right\}$ is $n_{s}$, $s\left(p_{1},p_{2},\ldots ,p_{is},\ldots ,p_{n_{s}}\right)$ is cubic spline interpolation curve for set $\left\{p_{1},p_{2},\ldots ,p_{is},\ldots ,p_{n_{s}}\right\}$; $PC=\left\{pc_{1},pc_{2},\ldots ,pc_{ic},\ldots ,pc_{n_{c}}\right\}$ is the reference candidate point set for parking location identification, the number of set *PC* is $n_{c}$, $\forall p_{is}\in PC$, and $n_{s}$ is considerably smaller relative to $n_{c}$.

The specific schematic diagram of intelligent parallel parking path optimization using cubic spline interpolation is expressed as follows.

Suppose there exists a point *s* in the parking region, its coordinate in the standard parking plane system is $\left(x_{s},y_{s}\right)$, and its angle with the horizontal axis is ∠β, obviously, $∠\beta =\arcsin(\frac{y_{s}}{\sqrt{{x_{s}}^{2}+{y_{s}}^{2}}})$. If the coordinate $\left(x_{s},y_{s}\right)$ is known, the conversion formula of the coordinate $\left(x_{r},y_{r}\right)$ of the actual scene parking plane system can be obtained as follows:

(3) $$\begin{array}{c} \begin{array}{c} x_{r}=\sqrt{{x_{s}}^{2}+{y_{s}}^{2}}\cdot \cos\left(∠\beta -∠\alpha \right)+x_{0}\\ y_{r}=\sqrt{{x_{s}}^{2}+{y_{s}}^{2}}\cdot \sin\left(∠\beta -∠\alpha \right)+y_{0} \end{array} \end{array}$$

Similarly, if the coordinate $\left(x_{r},y_{r}\right)$ is given, it’s easy to know the coordinate $\left(x_{s},y_{s}\right)$.

In the standard parking coordinate system, the set of decision variables is set as ${\left\{\begin{array}{c} \left(x\left(P_{2}\right),y\left(P_{2}\right)\right)\\ \ldots ,\ldots ,\\ \left(x\left(P_{2}\right),y\left(P_{2}\right)\right) \end{array}\right\}}^{T}$, and $x\left(P_{is}\right)$ and $y\left(P_{is}\right)$ are the horizontal and ordinate of the center point $P_{is}$ of the *is*-th fixed-point region in the standard parking coordinate system, respectively.

Parking experience is especially crucial. As can be seen from the above formulas, for the set of center points within a designated area, both the horizontal and vertical coordinate values gradually decrease during the parking maneuver until the vehicle reaches the parking position $p_{ns}$. However, in general, during the initial stage of parallel parking, the horizontal coordinate value decreases rapidly, while in the later stage, the vertical coordinate value decreases faster. Therefore, the entire driving process can be divided into a *x* decreasing zone and a *y* decreasing zone.

### 2.3. Novel Intelligent Vehicle Parking Path Optimization Model

Given a sampling period Δ*t*, by comparing the decreasing values of the horizontal and vertical coordinates ($\Delta x_{is}$ and $\Delta y_{is}$) between the current sampling position $p_{is}$ and the previous sampling position $p_{i_{s-1}}$, it is evident that the following relationship holds: $is>1,\Delta x_{is}>0,\Delta x_{is}>0$. Based on the above parking operation experiences, if these relationships hold, $\Delta x_{is}>\Delta y_{is}$, it indicates that the vehicle is still in the early stages of the parking process, which means it is in the *x* decreasing zone. On the contrary, if these relationships are invalid, the vehicle is in the *y* decreasing zone.

Generally, when a standard vehicle is parked in a standard garage, there exists a decreasing balance zone during the parking maneuver. The axis decreasing balance value of this decreasing balance zone can be set as a variable *δt*, which depends on the sampling period and the average parking speed. During the driving process, for the current sampling position $p_{is}$, if $|\Delta x_{is}-\Delta y_{is}|<\delta$, then it is considered that the intelligent vehicle is currently within the decreasing balance zone. Due to the obstacle avoidance requirement, it is necessary to consider whether the intelligent vehicle is far enough from the near corner point $P_{nt}$ of the garage roof at this time. In other words, the following constraints need to be met:

(4) $$\begin{array}{c} D\left(P_{is},P_{nt}\right)>0.5\cdot W_{C} \end{array}$$

where $D\left(A,B\right)$ is the linear distance between two position points (*A* and *B*); *WC* is the vehicle width.

For the decreasing balance zone, the definition of the mean obstacle avoidance distance within the decreasing balance zone is as follows.

Suppose that within the decreasing balance zone, sampling points are collected, and their set is denoted as $\left[P_{b1}\ldots P_{bis}\ldots P_{bns}\right]$, $DB=\frac{1}{bns}\sum_{bis=b1}^{bns}D\left(Pbis,Pnt\right)-0.5\cdot WC$.

For the mean value of obstacle avoidance distance in the decreasing balance zone, a smaller value indicates that driving in the initial stage of the parking process is better, making it easier to achieve a more satisfactory parking result.

So, the specific novel intelligent vehicle parking path optimization model is depicted as follows.

(5) $$\begin{array}{l} \min DB,L=\int_{0}^{T_{\max}}v\left(t\right)dt\approx \sum_{it=1}^{nt}\Delta L_{it}\\ S.T.\left\{\begin{array}{c} LT_{X,Y}=s\left(p_{1},p_{2},\ldots ,p_{is},\ldots ,p_{n_{s}}\right)\\ \Delta L_{it}\leq \Delta L_{\max}\&\Delta {∠}_{it}\leq \Delta {∠}_{\max}\\ \forall p_{is}\in P_{C}\\ \forall P_{g}\left(i\right)\notin \Omega_{c,it}\\ \forall P_{g}\left(i\right)\in \Omega_{g}\\ D\left(P_{\mathrm{bis}},P_{nt}\right)-0.5W_{C}>0 \end{array}\right. \end{array}$$

## 3. Improved Immune Moth–Flame Algorithm

### 3.1. Moth–Flame Optimization Algorithm

Moth–flame optimization (MFO) algorithm is a bio-inspired swarm intelligence proposed in 2015, inspired by the natural transverse orientation behavior of moths. Moths fly in a straight line in the natural environment by maintaining a fixed angle with the moonlight. However, when they mistakenly take an artificial light source as the moonlight, they will gradually approach the light source along a spiral trajectory. The MFO algorithm formally models this biologically grounded navigation mechanism. Within the algorithmic framework, “moths” represent candidate solutions to the optimization problem, while “flames” denote the set of optimal solutions identified up to the current iteration.

Let $X^{\left(t\right)}$ denote the population of moths at the *t*-th iteration, comprising *n* search individuals.

(6) $$\begin{array}{c} X^{\left(t\right)}=\left\{{\vec{x}}_{1},{\vec{x}}_{2},\ldots ,{\vec{x}}_{n}\right\}\in R^{\left(n\times d\right)} \end{array}$$

In this formulation, *n* signifies the overall count of moths in the population, while *d* indicates the number of dimensions characterizing the solution space. The set $F_{X}$ is used to encapsulate the fitness scores corresponding to the moth population.

(7) $$F_{X}=\left\{f\left({\vec{x}}_{i}\right)|{\vec{x}}_{i}\in X^{\left(t\right)}\right\}$$

The collection of optimal solutions (the flame) is denoted by Φ^(*t*)^.

(8) $$\begin{array}{c} \Phi^{\left(t\right)}=\left\{{\vec{\Phi }}_{1},{\vec{\Phi }}_{2},\ldots ,{\vec{\Phi }}_{n}\right\}\in R^{n\times d} \end{array}$$

The fitness scores of all flame individuals are encapsulated into the set $F_{\Phi }$.

(9) $$\begin{array}{c} F_{\Phi }=\left\{f\left({\vec{\phi }}_{i}\right)|{\vec{\phi }}_{i}\in \Phi^{\left(t\right)}\right\} \end{array}$$

The MFO algorithm operates through an iterative process governed by three fundamental computational operators.

(10) $$\begin{array}{c} A_{MFO}=\langle I_{nit},U_{pd},T_{erm}\rangle \end{array}$$

where $I_{nit}$ corresponds to the algorithm’s initialization operation, mainly manifested as: randomly generating a moth population within the preset solution space and determining the fitness score of each moth. The mapping relationship of this step is:

(11) $$\begin{array}{c} I_{nit}:\varphi \to \left\{X^{\left(t\right)},F_{X}\right\} \end{array}$$

$U_{pd}$ embodies the core positional update strategy employed by each moth during the optimization phase. During the optimization procedure, moths adjust their locations using a logarithmic spiral trajectory, which incorporates both their own current coordinates and the spatial distribution of the flame population. Following position adjustment, the fitness scores at the new location are computed. Whenever this newly obtained fitness score outperforms the corresponding flame, the flame’s location and fitness score are replaced accordingly. This step establishes a deterministic mapping between moths and flames.

(12) $$\begin{array}{c} U_{pd}:\left(X^{\left(t\right)},\Phi^{\left(t\right)}\right)\to X^{\left(t+1\right)} \end{array}$$

The position of each moth is iteratively adjusted using a logarithmic spiral trajectory as follows:

(13) $$\begin{array}{c} {\vec{x}}_{i}^{\left(t+1\right)}={∥{\vec{x}}_{i}^{\left(t\right)}-{\vec{\Phi }}_{j}^{\left(t\right)}∥}_{2}\cdot e^{\theta r}\cos\left(2\pi r\right)+{\vec{\varphi }}_{j}^{\left(t\right)} \end{array}$$

where ${\vec{x}}_{i}^{\left(t+1\right)}$ signifies the updated spatial position vector of the i-th moth, while ${\vec{\varphi }}_{j}^{\left(t\right)}$ indicates the location vector associated with the *j*-th flame. ${∥{\vec{x}}_{i}^{\left(t\right)}-{\vec{\Phi }}_{j}^{\left(t\right)}∥}_{2}$ denotes the Euclidean norm of the displacement vector between the *i*-th moth and the *j*-th flame; *θ* is a predefined constant governing the curvature of the logarithmic spiral; *r* is a uniformly distributed random variable in the interval $\left[-1,1\right]$; and $e^{\theta r}\cos\left(2\pi r\right)$ constitutes the logarithmic spiral function, which mathematically models the moth’s helical convergence trajectory toward the light source.

(14) $$\begin{array}{c} \eta \left(t\right)=round\left(N_{\max}-t\cdot \frac{N_{\max}-1}{T_{\max}}\right) \end{array}$$

where η(t) signifies the number of flames adjusted at iteration *t*; $N_{\max}$ stands for the upper limit on the flame count; *t* denotes the number of the current iteration count; and $T_{\max}$ specifies the total number of iterations allowed [21].

The stopping criterion, labeled $T_{erm}$, determines when the algorithm halts: execution concludes once either the desired level of solution accuracy is attained or the iteration number reaches $T_{\max}$. Meanwhile, the optimal solution identified during the search process is reported.

### 3.2. Immune Mechanism

The immune mechanism models the global optimal objective as an “antigen” and the individuals in the algorithm population as “antibodies”. In the standard iterative procedure of the MFO algorithm, overly rapid convergence of the population toward the current optimal solution severely compromises diversity, thereby resulting in the “premature convergence” phenomenon.

To tackle this problem, the present study incorporates a concentration-based selection approach rooted in artificial immune system principles, enabling adaptive control of population diversity via a similarity metric computed between individuals. Under this framework, the concentration of an antibody and its associated concentration probability are mathematically formulated as follows:

(15) $$\begin{array}{c} D\left({\vec{x}}_{i}\right)={\left(\sum_{k=1}^{n+\eta (t)}\left|f\left({\vec{x}}_{i}\right)-f\left({\vec{z}}_{k}\right)\right|+\varepsilon \right)}^{-1} \end{array}$$

(16) $$\begin{array}{c} P_{sel}\left({\vec{x}}_{i}\right)=\frac{D^{-1}\left({\vec{x}}_{i}\right)}{\sum_{k=1}^{n+\eta (t)}D^{-1}\left({\vec{z}}_{k}\right)}=\frac{\sum_{j=1}^{n+\eta (t)}\left|f\left({\vec{x}}_{i}\right)-f\left({\vec{z}}_{j}\right)\right|}{\sum_{k=1}^{n+\eta (t)}\sum_{j=1}^{n+\eta (t)}\left|f\left({\vec{z}}_{k}\right)-f\left({\vec{z}}_{j}\right)\right|} \end{array}$$

in Equations (15) and (16), $f(x_{i})$, $D(x_{i})$ and $P_{sel}\left({\vec{x}}_{i}\right)$ represent the fitness score, antibody concentration and concentration probability of the *i*-th individual in the moth swarm, respectively, where i=1,2,…,n+η(t).

Within the concentration selection framework, the immune system selectively enhances antibodies with greater killing power against the antigen while simultaneously down-regulating those with lower killing power and higher concentration. Introducing a concentration selection mechanism into the MFO algorithm can adaptively adjust the distribution of moths across the search space to achieve the goal of escaping the local optimum and seeking the global optimum.

### 3.3. Adaptive Inertia Coefficient

The inertia weight coefficient plays a decisive role in shaping the optimization behavior of swarm intelligence algorithms, effectively mediating the algorithm’s capacity between global exploration and local exploitation. A higher inertia weight strengthens the algorithm’s capacity for global exploration to aid in avoidance of local optima; in contrast, a lower value shifts the focus toward intensified local refinement to enhance precision in convergence. This study proposes inertia weight schemes to overcome two key limitations of the MFO: its sluggish convergence in the later stages of iteration and its tendency to yield insufficient accuracy solutions. Hence, a sine-based nonlinear decay function is adopted to govern the inertia weight’s decreasing across iterations to enhance the adaptability of the algorithm in different search stages.

The adaptive inertia weight coefficient is determined by the following expression:

(17) $$\begin{array}{c} \omega \left(t\right)={\left(1-\sin\frac{\pi t}{2T_{\max}}\right)}^{\mu } \end{array}$$

Consequently, the moth’s position update equation is modified after introducing the adaptive inertia weight coefficient as follows:

(18) $$\begin{array}{c} {\vec{x}}_{i}^{\left(t+1\right)}={∥{\vec{x}}_{i}^{\left(t\right)}-{\vec{\Phi }}_{j}^{\left(t\right)}∥}_{2}\cdot e^{\theta r}\cos\left(2\pi r\right)+\omega \left(t\right)\cdot {\vec{\Phi }}_{j}^{\left(t\right)} \end{array}$$

in Equations (17) and (18) define ω(t) as an adaptive inertia weight that adjusts dynamically across iterations; *θ* is a fixed parameter governing the logarithmic spiral trajectory (typically set to a constant 1 to maintain the standard spiral shape), while *r* is a stochastic variable drawn from a uniform distribution, and *μ* is the crucial regulating factor that dictates the decay rate of the adaptive inertia weight. By adjusting the value of *μ*, the decay rate of the inertia weight can be regulated to accommodate distinct search requirements: specifically, setting *μ* = 1 yields a standard sinusoidal decay; a value of *μ* < 1 retards the decay process, maintaining higher inertia for a prolonged duration to bolster global exploration; conversely, *μ* > 1 accelerates the decay, precipitating an earlier transition into local exploitation. During initial iterations, the relatively high value of ω(t) promotes global exploration across the solution space, to support diversity within the moth population. In contrast, its progressive attenuation in subsequent iterations intensifies local search activity, to enhance refinement optimization in approaching the global optimal region.

### 3.4. Elite Opposition-Based Learning Mechanism

Opposition-based learning (OBL) has been consistently demonstrated to enhance the global search capability of swarm intelligence algorithms [22]. It is grounded in the insight that, while probing an unknown search space, both a given solution and its oppositional likely to approach to the global optimum. Limiting the search to a single direction significantly increases the risk of missing promising areas that contain high-quality solutions.

To overcome the premature convergence of the MFO algorithm toward local optima during later stages of iteration, this study incorporates OBL mechanism. Once stagnation in population evolution is identified, the strategy employs dynamically updated reverse data within the algorithm’s current adaptive search boundary to generate a set of high-performing reverse candidate solutions, thereby enabling rapid escape from local optima and substantially improving both the algorithm’s capacity for global search and its precision in converging toward optimal solutions.

The opposition-based learning approach has been enhanced and mathematically articulated in Equation (19).

(19) $$\begin{array}{c} \left\{\begin{array}{c} {\tilde{x}}_{i,j}=\lambda \cdot \left(lb_{j}+ub_{j}\right)-x_{i,j}\\ {\tilde{x}}_{i,j},x_{i,j}\in \left[lb_{j},ub_{j}\right]\\ j=1,2,\ldots ,d \end{array}\right. \end{array}$$

where ${\tilde{x}}_{i,j}$ and $\;x_{i,j}$ stand for the elite opposition-based solution and the original solution, respectively, associated with the *i*-th individual’s position in the *j*-th dimension. $lb_{j}$ and $ub_{j}$ correspond to the minimum and maximum values admissible along the current dimension of the search space; $\lambda \in \left[0,1\right]$ is an iteratively refined generalization parameter generated stochastically per iteration. This dynamic generation strategy deliberately avoids fixed empirical values, thereby continuously promoting the randomness and diversity of the reverse solution generation; *d* denotes the number of dimension that constitute the solution domain.

If the value along the *j*-th dimension surpasses the designated limit, the corrective procedure outlined in Equation (20) is invoked.

(20) $$\begin{array}{c} {\tilde{x}}_{i,j}=lb_{j}+\xi \cdot \left(ub_{j}-x_{i,j}\right) \end{array}$$

let $\xi \in \left[0,1\right]$ be the random remapping factor.

This correction mechanism utilizes the relative position information between the original solution and the boundary to remap the overflow solution back into the safe region. Hence, this approach not only maintains the rationality of the population distribution but also preserves spatial features inherent to the initial solution, thereby further contributing the robustness of the algorithm.

## 4. Research Methodology

### 4.1. Research Objectives and Hypotheses

The primary objective of this study is to formulate an intelligent vehicle parking path optimization model utilizing cubic spline interpolation and to develop the IIMFO algorithm to significantly elevate the computational efficiency and spatial precision of autonomous parking trajectory generation. We hypothesize that a path generation framework driven by cubic spline interpolation effectively translates the complex trajectory optimization problem into a computationally efficient, kinematically feasible solution with continuous curvature. Meanwhile, integrating an artificial immune concentration selection mechanism, a nonlinear adaptive inertia weight (where *μ* is a regulating factor), and an elite OBL mechanism into the standard MFO architecture will dynamically enrich population diversity, yielding substantial improvements in global search efficacy and convergence accuracy.

### 4.2. Experimental Setup and Validation Scenarios

To rigorously validate the proposed model, the IIMFO algorithm was compared against established baseline algorithms, including MFO [12], PSO, WOA, and GA. The research methodology was structured across three continuous validation platforms. Initially, standardized benchmark testing was conducted using De Jong’s Function, Schaffer’s Function, and DTLZ1 to theoretically assess the algorithm’s convergence precision. Subsequently, computer-based simulations were established to support intelligent trajectory optimization. This study established a coordinate system as follows: the x-axis was aligned with the lower boundary of the garage; the y-axis was oriented along the garage’s vertical sideline situated farthest from the vehicle and perpendicular to the x-axis; the coordinate origin was defined as the point at which the x-axis and y-axis intersect. Two trajectory optimization scenarios were implemented using the Volkswagen UP (Volkswagen, Wolfsburg, Germany) and Honda XR-V (Honda Motor Co., Ltd., Tokyo, Japan). In the experimental setup, the initial coverage region was positioned such that its edge remained parallel to and 2.2 m away from the garage boundary line located at the corner. The parking setup for the Volkswagen UP was specified as a dedicated parking area of 5.0 m by 2.5 m, with the vehicle body measuring 3.5 m in length and 1.7 m in width. The parking setup for the Honda XR-V allocated the same 5.0 m by 2.5 m area, while the vehicle body measured 4.4 m in length and 1.8 m in width.

Finally, owing to the difficulty in realizing a perfectly intelligent experimental setting, this study employed a semi-automatic configuration for empirical validation. Under this configuration, a rearview camera equipped with a computationally optimized parking trajectory functions as a visual aid, while the driver engaged in the vehicle’s semi-automatic driving functionality. The optimized parking route is the principal driving factor in the parking process; nonetheless, driver expertise remains essential.

The experimental site for semi-automatic testing was the No. 148 parking space situated in the Xinghai Square Shell Museum, Dalian, China. The evaluation was conducted using the Toyota LeiLing Shuangqing 185T Sportline (GAC Toyota Motor Co., Ltd., Guangzhou, China) as the test vehicle, defined by a parking zone measuring 5.0 m by 2.5 m, a vehicle body of 4.7 m × 1.8 m, initially positioned 1.0 m away from the adjacent garage sideline, with the horizontal separation between the originally designated coverage zone and the closest vertex of the garage measuring 2.2 m.

To execute the automated parking tasks within these physical spatial scenarios, the experimental vehicle is equipped with a comprehensive Automatic Reverse Parking (ARP) system. The ARP architecture integrates real parking data acquisition sensors, a spatial feasibility judgment device, a reference trajectory optimizer, a tracking controller, dedicated stopping and emergency braking mechanisms, an imaging system, and an upper monitoring computer. During the parking procedure, onboard sensors dynamically feed data for feasibility analysis and tracking control. The optimizer then transmits the optimized trajectory simultaneously to the parking controller and the upper computer. This dual-transmission not only guarantees accurate tracking references but also provides the driver with a comprehensive real-time view to ensure operational safety. To guarantee execution efficiency, the limit times for data acquisition, feasibility judgment, trajectory optimization, tracking control, emergency braking, and final parking stop were strictly constrained to 3.5 s, 1.5 s, 12 s, 25 s, 0.4 s, and 1.5 s, respectively.

**Figure 2:**
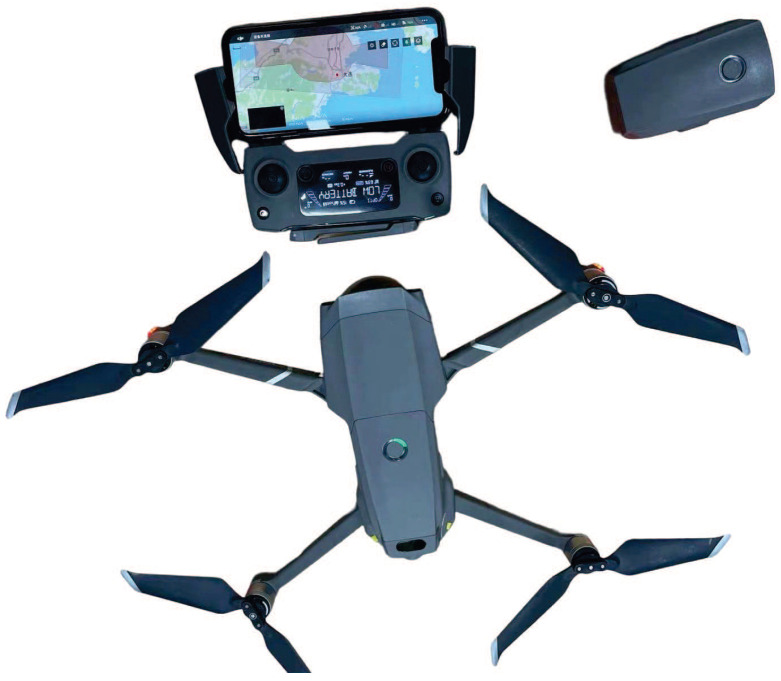
Physical structural configuration of Mavic 2 DJI’s unmanned aerial vehicle.

The underlying hardware and safety configurations were rigorously established to handle these tasks. For the core computational hardware, two MPC555LFMZP40 microcontrollers (NXP Semiconductors, Eindhoven, The Netherlands)were deployed to serve as the trajectory optimizer (handling data acquisition, feasibility judgment, and trajectory optimization) and the tracking controller (executing tracking control, emergency stops, and final parking stops). Concurrently, two DSP28335 chips (Texas Instruments, Dallas, TX, USA) were utilized for the auxiliary control unit (ACU, managing the air-conditioner, overload protection, communication, etc.) and the actuator power unit (APU, controlling the breaker, steering gear, braking/dynamical systems, and gear control). The upper monitoring computer, a MacBook Pro (2016 Core i5 @ 2.9 GHz, Apple Inc., Cupertino, CA, USA) secured to the vehicle’s interior mobile phone holder, established communication with the vehicle via Bluetooth. Given the critical security nature of early-stage ARP technology, the system features an immediate emergency brake triggered upon risk perception. Furthermore, the experiment was conducted under strict multi-layered human supervision: a driver holding a C2 license served as both the in-vehicle safety officer and manual control corrector, and a ground safety inspector maintained constant communication with the driver. To capture the macroscopic kinematics of the entire process, this work utilized the Mavic 2 DJI’s unmanned aerial vehicle (UAV) (DJI, Shenzhen, China) as the aerial imaging system. Its structural configuration and key hardware components are illustrated in Figure 2.

### 4.3. Empirical Parameter Configurations

Before proceeding with the comprehensive benchmark evaluations, an empirical sensitivity confirmation concerning the smoothing control parameter *μ* was necessitated to strictly validate the theoretical assumptions formulated in Section 3.3. Corroborating the prior theoretical analysis, preliminary simulation trials with *μ* in {0.5,1.0,1.5,2.0} conclusively demonstrated that *μ* = 1.0 effectively averts both the over-exploration observed at lower values (*μ* < 1) and the premature exploitation triggered at higher values (*μ* > 2.0). Consequently, in strict empirical alignment with the established theoretical premise, *μ* = 1.0 was rigidly locked as the standardized default configuration for all ensuing experimental evaluations.

### 4.4. Evaluation Metrics

To conduct an objective performance comparison among the proposed IIMFO and baseline algorithms, specific evaluation metrics were defined based on established optimization evaluation practices. For the single-objective benchmarks, the optimal minimum function value was employed to assess the algorithm’s ultimate convergence accuracy toward the true theoretical minimum, while the mean and standard deviation quantify algorithmic stability against stochastic uncertainties [12]. Furthermore, for the high-dimensional DTLZ1 function, the IGD metric was utilized to simultaneously evaluate the convergence capability and the robust scalability in escaping local traps within high-dimensional multi-objective search spaces [17]. In the intelligent parking optimization model, the length of the parking path trajectory, defined as the definite integral of parking velocity over the time period from the parking begin to the parking termination time, served as the primary physical metric for evaluating spatial optimization efficiency.

## 5. Experimental Results and Discussion

### 5.1. Convergence and Robustness Evaluation on Benchmark Functions

The comparative simulation outcomes were obtained using MATLAB R2022b (MathWorks, Natick, MA, USA) and are summarized below:

(I) De Jong’s function-whose optimal minimum is known to be exactly zero.

**Figure 3:**
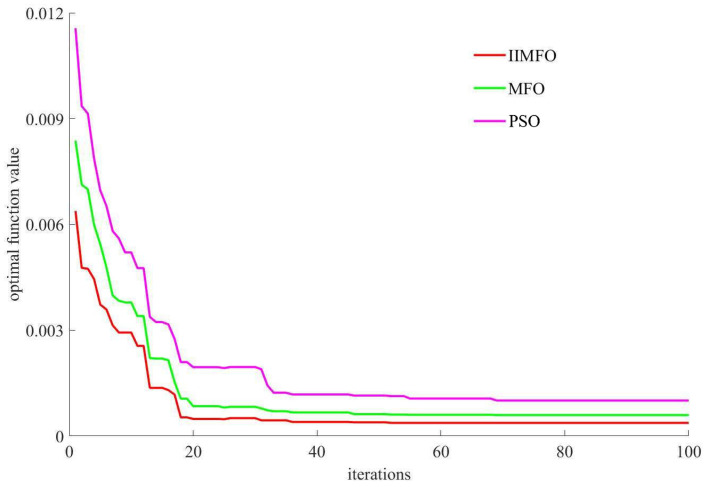
Iterative convergence curves of solution quality for 3-dimensional De Jong’s function.

To quantitatively assess the convergence precision, a detailed performance evaluation on the 3-dimensional (3D) De Jong benchmark function (whose theoretical optimal minimum is exactly zero) was conducted. The iterative convergence curves and the statistical outcomes are presented in Figure 3 and Table 1 respectively.

Figure 3 and Table 1 demonstrate that the IIMFO algorithm achieves superior performance relative to both MFO and PSO, and its optimal minimum function value is $\left(x_{1},x_{2}\right)=\left(0.9923,0.9941\right)$, the optimal function value is $F\left(x_{1},x_{2}\right)=3.8\times 10^{-4}$.

(II) The 3-dimensional (3D) Schaffer function (whose theoretical optimal minimum is 0.0), it is given as follows.

**Figure 4:**
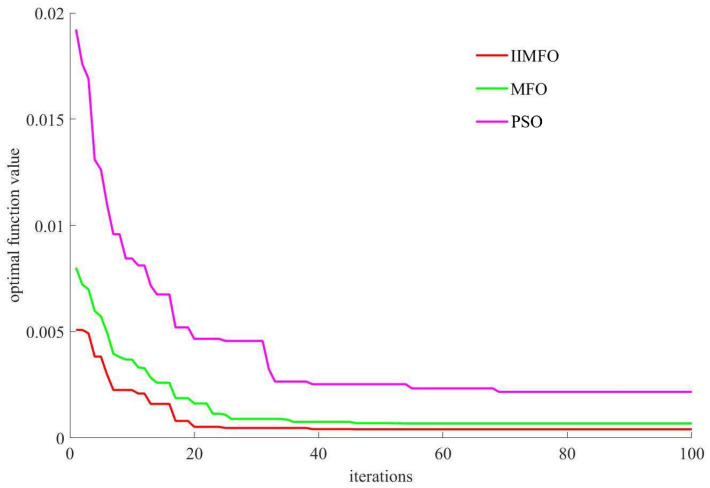
Iterative convergence curves of solution quality for 3-dimensional Schaffer function.

The corresponding iterative convergence curves and quantitative statistical results for the 3D Schaffer function are illustrated in Figure 4 and Table 2, respectively.

Figure 4 and Table 2 demonstrate that the IIMFO algorithm achieves superior performance relative to both MFO and PSO, and its optimal minimum function value is $\left(x_{1},x_{2}\right)=\left(3.2\times 10^{-4},2.4\times 10^{-4}\right)$, the optimal function value is $F\left(x_{1},x_{2}\right)=4.0\times 10^{-4}$.

To further validate the robustness and scalability of the algorithms against higher-dimensional complexities, an additional test was conducted using the DTLZ1 benchmark function. The specific analytical formulation of the 3-dimensional (3D) DTLZ1 function is expressed as follows:

(21) $$\left\{\begin{array}{c} \left\{\begin{array}{c} f_{1}\left(x\right)=\frac{1}{2}x_{1}x_{2}\left(1+g\left(x\right)\right)\\ f_{2}\left(x\right)=\frac{1}{2}x_{1}\left(1-x_{2}\right)\left(1+g\left(x\right)\right) \end{array}\right.\\ g\left(x\right)=100\left(10+\sum_{i=3}^{n}{\left(x_{i}-0.5\right)}^{2}-\cos\left(20\pi \left(x_{i}-0.5\right)\right)\right) \end{array}\right.x={\left(x_{1},x_{2},\ldots ,x_{n}\right)}^{T}\in {\left[0,1\right]}^{n}$$

where $x={\left(x_{1},x_{2},\ldots ,x_{n}\right)}^{T}$ represents the n-dimensional decision variable vector, with each individual element $x_{i}$ bounded within the continuous interval [0, 1]. $f_{1}\left(x\right)$ and $f_{2}\left(x\right)$ denote the objective functions to be evaluated. The term g(x) acts as a highly complex landscape distance function, which deliberately introduces a massive number of local optima. This specific formulation is utilized to rigorously challenge the optimization algorithm’s convergence capability and its robust scalability in escaping local traps within high-dimensional search spaces.

**Figure 5:**
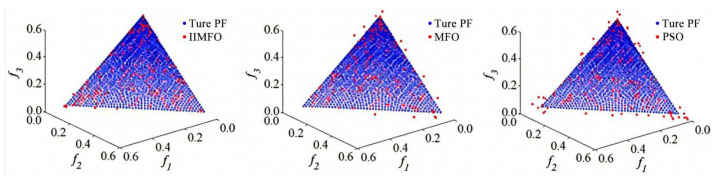
Iterative convergence curves of IGD values for 3-dimensional (3D) DTLZ1 function.

The specific evaluation outcomes for the 3D DTLZ1 function, assessed utilizing the Inverted Generational Distance (IGD) metric, are visually and quantitatively presented in Figure 5 and Table 3.

As demonstrated by Figure 5 and Table 3, the proposed IIMFO algorithm achieves the most superior performance compared to the other algorithms. Its optimal IGD value is precisely 2.46 × 10^−3^. This explicitly proves that the improved strategy maintains strong robust search capabilities even when dealing with the complex multimodal landscape of the DTLZ1 function.

### 5.2. Optimization Results of Reference Trajectories for Real-Vehicle Experiments

The parking path optimization methods employed to generate the reference trajectories in this comparative study include the improved immune moth–flame optimization (IIMFO) algorithm proposed in this paper, alongside four baseline algorithms: the original moth–flame optimization (MFO), the whale optimization algorithm (WOA), the particle swarm optimization (PSO), and the genetic algorithm (GA).

As detailed in Section 4.2, the reference trajectory optimization evaluations are conducted under two representative parallel parking scenarios: a small car (Volkswagen UP, Volkswagen, Wolfsburg, Germany; 3.5m×1.7m) and an SUV (Honda XR-V, Honda Motor Co., Ltd., Tokyo, Japan; 4.4m×1.8m). For both configurations, the standard garage dimensions are consistently set to 5.0m×2.5m. In the established Cartesian coordinate system (where the origin O is clearly marked in the figures), the initial coverage region is positioned such that its edge remains parallel to and 2.2 m away from the garage boundary line located at the corner.

**Figure 6:**
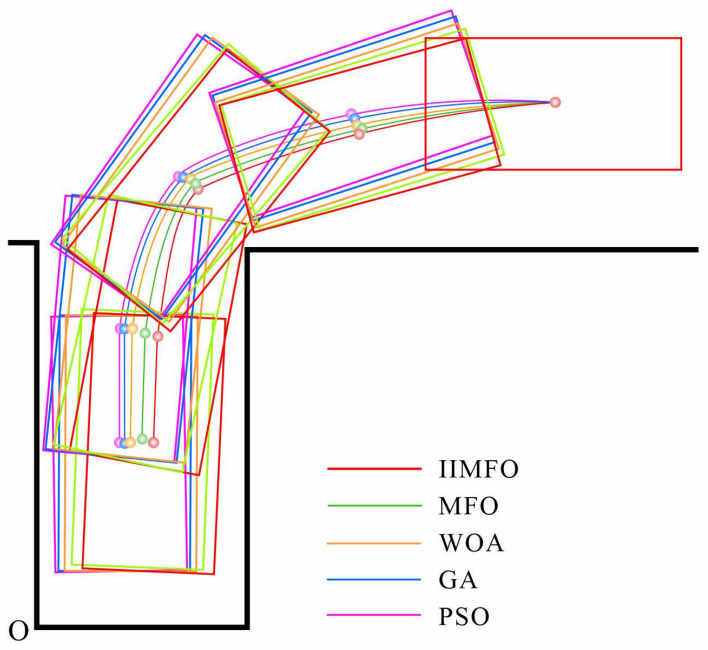
Operation parking trajectories for Volkswagen UP.

**Figure 7:**
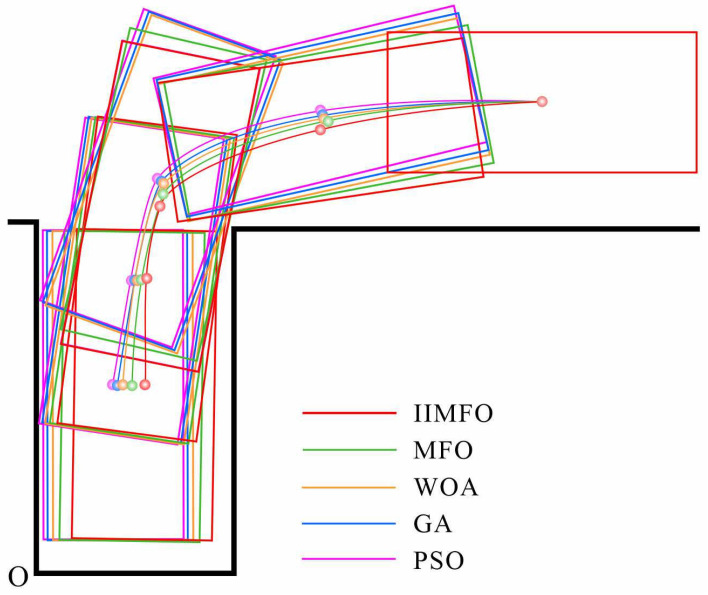
Operation parking trajectories for Honda XR-V.

Figure 6 and Figure 7 visually illustrate the optimized reference parking trajectories generated by the five algorithms for the Volkswagen UP and Honda XR-V scenarios. The associated quantitative outcomes, detailing the trajectory lengths and final parking location reference points, are comprehensively summarized in Table 4 and Table 5.

From the perspective of quantitative optimization, IIMFO demonstrates exceptional generality and spatial efficiency across both vehicle dimensions. As illustrated in Figure 6 and Table 4, IIMFO achieves the shortest reference trajectory of 9.44 m in the Volkswagen UP scenario. This represents a significant reduction in path length compared to the worst-performing PSO (10.99 m) and GA (10.78 m). Furthermore, it still secures distinct improvements over WOA (10.32 m) and the standard MFO (9.79 m).

As illustrated in Figure 7 and Table 5, IIMFO consistently surpasses all comparative algorithms in the Honda XR-V SUV scenario, where spatial constraints are more stringent, achieving the optimal trajectory of 9.36 m. Demonstrating consistent superiority, this translates to noticeable path reductions against PSO (10.80 m) and GA (10.65 m), alongside steady improvements over WOA (10.09 m) and MFO (9.70 m).

As the vehicle maintains an approximately steady speed while parking, such consistent trajectory reductions across different scenarios directly translate to less parking time, thereby significantly enhancing the overall user experience.

When parking without collision the side lines of the vehicle garage, the IIMFO-generated parking trajectory demonstrates enhanced smoothness and reduced path length relative to those produced by MFO, WOA, GA, and PSO. Notably, compared to the traditional GA method, which yielded the longest trajectories in both scenarios, the IIMFO significantly shortens the parking trajectory, thereby achieving the optimal parking. The results indicate that IIMFO algorithm achieves enhanced optimization capability, thereby supporting more efficient intelligent trajectory planning.

### 5.3. Semi-Automatic Experimental Results

**Figure 8:**
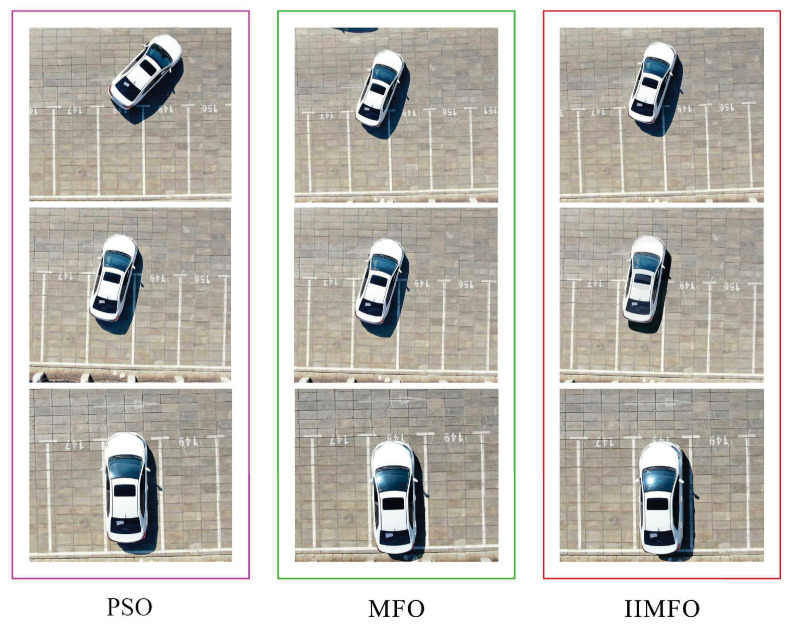
Captured key fixed reference points images for semi-automatic parking progression.

This study conducted the real-world comparison of a semi-automatic parking system utilizing intelligent optimization trajectory. The real-world comparison experiment was carried out on 10 August 2025, under quiescent and cloud-free atmospheric conditions to ensure consistent UAV imaging performance. Three key control points were acquired using the DJI Mavic 2 Pro UAV (DJI, Shenzhen, China). The spatial location and acquisition context of these control points collected during the semi-automatic parking progression are presented in Figure 8.

As can be seen from the captured key fixed reference points images for semi-automatic parking progression using Toyota LeiLing Shuangqing 185T, compared with MFO and PSO, under the premise of without collision about side line of vehicle garage during parking process, the semi-automatic parking performance delivered by the IIMFO algorithm shows marked improvement. Hence, it indicates that IIMFO is a well-designed optimization method, endowed with high search efficiency and strong adaptability, thus offering superior effectiveness in tackling intelligent trajectory planning tasks.

### 5.4. Overall Performance Comparison Summary

To provide a centralized and comprehensive evaluation of the analyzed algorithms across all testing dimensions, a summarized comparison is presented in Table 6. The table synthesizes algorithmic characteristics, mathematical benchmark performances (encompassing low-dimensional convergence precision and high-dimensional robustness), simulation outcomes (trajectory length minimization), and adaptability in real-world semi-automatic experiments.

As intuitively illustrated in Table 6, conventional approaches such as GA and WOA exhibit pronounced limitations when navigating complex parking constraints, yielding the longest trajectory paths and demonstrating vulnerability to local optima. Although the original MFO algorithm presents acceptable baseline performance, it lacks the requisite self-adaptive refinement capabilities in high-dimensional or highly constrained environments. In stark contrast, the proposed IIMFO framework consistently ranks highest across all quantitative and qualitative evaluation metrics. By synergistically integrating artificial immune concentration selection, nonlinear adaptive inertia weights, and the elite OBL mechanism, IIMFO successfully bridges the gap between theoretical global optimization capacity and practical autonomous parking deployment, ensuring the shortest path lengths and superior trajectory smoothness.

### 5.5. Discussion the Hierarchical Parking Framework for AI-Defined Vehicles

To broaden the impact of this study and align it with the emerging paradigm of fully autonomous urban mobility, it is essential to contextualize the proposed IIMFO algorithm within a comprehensive intelligent parking ecosystem. The current problem formulation operates under the premise that the target parking space is predefined, focusing predominantly on the localized kinematic trajectory planning required to reach that space. However, in practical deployment scenarios, an AI-defined vehicle must first resolve a higher-level antecedent challenge: determining exactly where to park.

This macro-level decision-making process, known as online location planning, requires the vehicle to dynamically select an optimal parking site—potentially miles away from its current destination—based on a multitude of dynamic factors such as real-time space availability, pricing fluctuations, and future trip itineraries. Recent advancements have highlighted the criticality of this antecedent step. For instance, Zheng et al. [23] systematically investigated the online location planning problem for AI-defined vehicles, proposing a joint optimization framework that addresses both order serving and spatio-temporal heterogeneous model fine-tuning.

To bridge the gap between macroscopic dispatching and microscopic execution, we outline a hierarchical autonomous parking framework. In this two-tier architecture, a high-level AI planner first utilizes real-time data streams and occupancy prediction models to select the globally optimal parking location. Once the specific destination is assigned, the IIMFO algorithm proposed in this study is activated as the low-level optimizer, taking over precise vehicle control to generate a collision-free, continuous-curvature trajectory for the final approach.

Furthermore, the “online” nature of this macro-level location planning introduces the critical requirement for rapid, adaptive decision-making under high uncertainty. While the proposed IIMFO is currently deployed as a kinematic trajectory optimizer, its foundational optimization principles—particularly its robust search capabilities and elite opposition-based learning for escaping local optima in complex spaces—exhibit significant potential for extension into this higher-level dynamic spatial selection. In future developments, the dynamic location selection problem could be formulated as a sequential decision-making process. By coupling the robust heuristic search mechanisms of IIMFO with advanced paradigms such as reinforcement learning (RL), the vehicle could adaptively navigate spatio-temporal uncertainties to identify optimal parking targets. Such an integration not only elevates the proposed IIMFO beyond a standalone maneuver planner but also establishes this work as a foundational methodology supporting the broader vision of autonomous urban navigation and intelligent parking ecosystems.

## 6. Conclusions

To overcome the shortcomings of conventional optimization approaches in intelligent trajectory control systems, this study establishes an intelligent parking path planning model leveraging cubic spline interpolation. Additionally, an improved immune moth–flame optimization (IIMFO) algorithm is introduced to enhance the efficiency and accuracy of path generation for autonomous vehicles. Given the identified deficiencies of the conventional moth–flame optimization (MFO) algorithm, this study integrates multiple modification strategies. The primary innovation contributions of this paper are outlined below:

(I) Key contribution to intelligent parking path planning: targeting the limitations of conventional trajectory optimization approaches—specifically, excessive path length and insufficient smoothness in the generated tracking trajectories—this study develops a cubic spline-based path planning framework for intelligent parking systems.
(II) Key contribution of the MFO algorithm: to meet the requirements of intelligent trajectory planning for intelligent parking systems and to resolve the inefficiency issues inherent in classical optimization methods, this study develops the IIMFO algorithm specifically for parking path optimization. The proposed enhancements aim to improve the algorithm’s ability to maintain diversity in the search process and avoid premature convergence. Specifically, the algorithm is enhanced to achieve these goals through three crucial modifications: (1) integrate an immunity strategy into the iterative scheme to reconfigure the spatial layout of moths, thereby overcoming the situation of getting stuck in a local optimum and progressively approaching the global optimum; (2) propose a dynamically adjusted inertia weight that gradually diminishes during the optimization process to improve the algorithm’s ability to explore the global search space; (3) incorporate an OBL strategy to elevate the solution quality of elite individuals during the optimization process.

Benchmark function evaluations confirm that IIMFO markedly enhances the MFO algorithm’s convergence rate, iterative solution accuracy, and overall stability. Moreover, comparative analyses encompassed both computational simulations and semi-automatic experimental validation under intelligent trajectory optimization, revealing that the IIMFO optimization effect has significantly improved in deployment scenarios.

Although the IIMFO algorithm demonstrates excellent efficiency and accuracy in optimizing intelligent vehicle parking trajectories, this study still presents certain limitations:

(I) Regarding parameter sensitivity and computational overhead, the nonlinear adaptive inertia weight relies heavily on the empirical setting of the regulating factor *μ*. Additionally, the artificial immune concentration mechanism requires extensive similarity calculations, significantly increasing iterative time complexity. Implementing lightweight surrogate models and online self-calibration is crucial for future dynamic adaptability.
(II) At the multi-objective optimization level, the model primarily targets path length and initial obstacle avoidance. Lacking explicit optimization for the steering angle change rate and energy efficiency, it may sacrifice ride comfort under extreme conditions. Integrating comprehensive multi-objective optimization algorithms is vital to coordinate diverse vehicle dynamics.
(III) Regarding the macroscopic deployment framework, the current problem formulation operates under the premise that the target parking space is predefined, focusing exclusively on localized kinematic trajectory planning. However, in the emerging paradigm of fully autonomous urban mobility, an AI-defined vehicle must initially resolve the antecedent macro-level challenge of online location planning. This involves dynamically selecting an optimal parking site from numerous potential locations based on real-time availability, pricing, spatial-temporal heterogeneity, and future trip demands [23]. Therefore, in future work, integrating our micro-level IIMFO trajectory planning model with macroscopic online location planning frameworks for AI-defined vehicles will represent a crucial transition toward fully autonomous and intelligent mobility.

## Figures and Tables

**Table 1:** Quantitative outcomes obtained across different optimization methods on 3-dimensional De Jong’s function.

Algorithm	$\left(x_{1},x_{2}\right)$	$F(x_{1},x_{2})$
IIMFO	0.9923, 0.9941	$3.8\times 10^{-4}$
MFO	0.9796, 0.9809	$5.9\times 10^{-4}$
PSO	0.9791, 0.9818	$1.2\times 10^{-3}$

**Table 2:** Quantitative outcomes obtained across different optimization methods on 3-dimensional Schaffer function.

Algorithm	$\left(x_{1},x_{2}\right)$	$F(x_{1},x_{2})$
IIMFO	$3.2\times 10^{-4},\;2.4\times 10^{-4}$	$4.0\times 10^{-4}$
MFO	$5.5\times 10^{-4},\;4.0\times 10^{-4}$	$6.8\times 10^{-4}$
PSO	$1.6\times 10^{-3},\;1.4\times 10^{-3}$	$2.2\times 10^{-3}$

**Table 3:** Quantitative IGD outcomes obtained across different optimization methods on the 3-dimensional (3D) DTLZ1 function.

Optimization Algorithm	IGD Value
IIMFO	$2.46\times 10^{-3}$
MFO	$6.72\times 10^{-3}$
PSO	$9.29\times 10^{-3}$

**Table 4:** Associated outcomes about trajectory optimization for Volkswagen UP.

Algorithm	Preference Point Set for Parking Location (m)	Trajectory Length (m)
IIMFO	(1.36,2.38), (1.32,3.83), (1.95,5.77), (4.07,6.49)	9.44
MFO	(1.20,2.49), (1.29,3.85), (1.91,5.82), (4.12,6.63)	9.79
WOA	(1.05,2.44), (1.09,3.93), (1.88,5.84), (3.98,6.67)	10.32
GA	(0.97,2.39), (0.97,3.93), (1.80,5.91), (3.94,6.78)	10.78
PSO	(0.92,2.41), (0.93,3.91), (1.77,5.95), (3.90,6.83)	10.99

**Table 5:** Associated outcomes about trajectory optimization for Honda XR-V.

Algorithm	Preference Point Set for Parking Location (m)	Trajectory Length (m)
IIMFO	(1.46,2.19), (1.54,4.24), (1.76,5.80), (3.87,6.44)	9.36
MFO	(1.26,2.20), (1.46,4.22), (1.69,5.91), (3.95,6.52)	9.70
WOA	(1.16,2.23), (1.40,4.20), (1.64,5.96), (3.91,6.54)	10.09
GA	(1.12,2.18), (1.37,4.21), (1.65,5.99), (3.90,6.58)	10.65
PSO	(1.09,2.20), (1.35,4.21), (1.63,6.02), (3.89,6.63))	10.80

**Table 6:** Comprehensive performance comparison of the evaluated algorithms.

Algorithm	Core Algorithmic Mechanisms	Low-Dimensional Benchmark Accuracy	High-Dimensional Robustness	Path Length Performance	Real-World Adaptability & Trajectory Smoothness
IIMFO	Immune concentration, adaptive inertia weight, elite OBL	Excellent (Highest precision)	High (Strong escape from local optima)	Shortest (9.44 m/9.36 m)	High (Continuous curvature, precise automated parking)
MFO	Standard logarithmic spiral search	Good	Moderate	Moderate (9.79 m/9.70 m)	Moderate (Prone to premature convergence)
WOA	Bubble-net attacking strategy	Fair	Fair	Long (10.32 m/10.09 m)	Moderate
GA	Genetic crossover and mutation	Weak	Weak	Longest (10.78 m/10.65 m)	Low (Highest computational cost and path length)
PSO	Swarm intelligence position and velocity update	Good	Moderate	Longest (10.99 m/10.80 m)	Low

## Data Availability

The original contributions presented in this study are included in the article. Further inquiries can be directed to the corresponding author.

## Abbreviations

The following abbreviations are used in this manuscript:

IIMFO	improved immune moth–flame optimization
MFO	moth–flame optimization
